# Acceptability, feasibility, equity and resource use for prenatal screening for chlamydia and gonorrhea: A systematic review

**DOI:** 10.14745/ccdr.v50i78a03

**Published:** 2024-07-24

**Authors:** Shamila Shanmugasegaram, Ulrick Auguste, Annie Fleurant-Ceelen, Stacy Sabourin, Annie-Claude Labbé, Jared Bullard, Gina Ogilvie, Mark H Yudin, Nancy Santesso

**Affiliations:** 1 Centre for Communicable Diseases and Infection Control, Public Health Agency of Canada, Ottawa, ON; 2 Hôpital Maisonneuve-Rosemont, Montréal, QC; 3 Université de Montréal, Montréal, QC; 4 University of Manitoba, Winnipeg, MB; 5 British Columbia Women’s Hospital and Health Centre, Vancouver, BC; 6 University of British Columbia, Vancouver, BC; 7 St. Michael’s Hospital, Toronto, ON; 8 University of Toronto, Toronto, ON; 9 McMaster University, Hamilton, ON

**Keywords:** chlamydia, gonorrhea, pregnancy, antenatal, prenatal, screening, testing, acceptability, feasibility, equity, resource use

## Abstract

**Background:**

A systematic review on acceptability, feasibility, equity and resource use was conducted as part of updating recommendations from the Public Health Agency of Canada on prenatal screening for *Chlamydia trachomatis* (CT) and *Neisseria gonorrhoeae* (NG).

**Methods:**

Information sources, including MEDLINE® All, Embase and Cochrane CENTRAL (January 2003–January 2021) electronic databases were searched for studies that assessed acceptability, feasibility, equity and resource use of screening for CT or NG in pregnant persons aged ≥12 years. The Risk of Bias Assessment Tool for Non-Randomized Studies was used for quality assessment and a narrative synthesis was prepared.

**Results:**

Of the 1,386 records identified, nine observational studies (approximately 5,000 participants) and three economic evaluations met the inclusion criteria. In general, pregnant persons and healthcare providers accepted screening. Most pregnant persons and partners supported universal testing for CT. Pregnant persons preferred non-invasive sampling methods. Inequities in feasibility (accessibility to screening) exist in certain populations. Studies have shown that targeted screening can miss cases. Screening all pregnant persons for CT has net cost savings compared to no screening. Limitations include not identifying eligible literature on acceptability of prenatal screening for NG among partners of pregnant persons and some studies with increased risk populations that restrict the generalizability of the findings highlighting areas for future research.

**Conclusion:**

Prenatal screening for CT and NG is generally acceptable among pregnant persons and healthcare providers. Evidence has shown that targeted screening can miss cases. The findings were included when updating PHAC’s recommendations on prenatal screening for CT and NG. This work was presented at the Society of Obstetricians and Gynaecologists of Canada’s 2024 Annual Clinical and Scientific Conference in Edmonton, Alberta.

## Introduction

In Canada, *Chlamydia trachomatis* (CT) and *Neisseria gonorrhoeae* (NG) are the most common reported sexually transmitted infections (STIs), with rates markedly increasing between 2010 and 2019 (CT, 33.1% and NG, 181.7%) ((1)). In 2010, 94,716 cases of CT and 11,381 cases of NG were reported in Canada, corresponding to rates of 278.5 and 33.5 per 100,000 population, respectively (1,2). In 2019, 139,386 cases of CT and 35,443 cases of NG were reported in Canada, corresponding to rates of 370.8 and 94.3 per 100,000 population, respectively ((1,2).

*Chlamydia trachomatis* and NG infections are often asymptomatic in females and can go undetected. In pregnant women/pregnant individuals (PWPI), this can lead to adverse outcomes. If the birthing parent has not received an effective treatment during the perinatal period, infection can potentially be transmitted to the neonate during delivery and lead to adverse neonatal health outcomes. If left untreated, CT in the birthing parent carries a 30%–50% risk of the neonate developing ophthalmia neonatorum and 10%–20% risk of developing CT pneumonia ((3)). *Neisseria gonorrhoeae* infection in the birthing parent carries a 30% risk of the neonate developing gonococcal ophthalmia neonatorum (4,5). Potential consequences of ophthalmia neonatorum include permanent visual impairment. There is lack of national surveillance information on gonococcal ophthalmia neonatorum, chlamydial ophthalmia neonatorum and neonatal pneumonia cases.

In 2010, the Public Health Agency of Canada (PHAC) recommended that all pregnant women should be evaluated for STI risk factors prior to and during pregnancy. Any woman with ongoing risk factors for STI acquisition during pregnancy should be considered for rescreening each trimester (6). In 2010, PHAC also recommended screening for CT early in pregnancy. Repeat screening should be performed in the third trimester for women at continuing risk for STI acquisition ((6)). In 2016 (reaffirmed in 2021), the Canadian Paediatric Society stated, “Neonatal ocular prophylaxis with erythromycin, the only agent currently available in Canada for this purpose, may no longer be useful and, therefore, should not be routinely recommended” (7). Variation in practice exists with regard to offering neonatal ocular prophylaxis to prevent ophthalmia neonatorum. Evidence shows that approximately 15%–22% of PWPI are not being screened for CT and NG (8–10). Screening and testing for these infections could help prevent adverse pregnancy and neonatal outcomes.

Given the increasing rates of reported cases of CT and NG in the general population and suboptimal rates of prenatal screening for CT and NG in Canada ((8–10)), the National Advisory Committee on Sexually Transmitted and Blood-Borne Infections (NAC-STBBI) reviewed and updated PHAC’s recommendations on prenatal screening for CT and NG. Canada's Drug Agency (CDA-AMC), formerly Canadian Agency for Drugs and Technologies in Health (CDA-AMC) conducted a health technology assessment (HTA) ((11)). The main objective of PHAC’s systematic review was to search, identify and synthesize relevant literature on acceptability, feasibility, equity and resource use on prenatal screening for CT and NG to support updating of the PHAC recommendations based on the Grading of Recommendations Assessment, Development and Evaluation (GRADE) approach ((12–14)) (*unpublished document, Shanmugasegaram S/Public Health Agency of Canada, Methods Manual for the Public Health Agency of Canada Sexually Transmitted and Blood-Borne Infections Recommendations, 2019*).

## Methods

According to the GRADE approach, the determinants of the strength and direction of guideline recommendations include acceptability among stakeholders, feasibility of the intervention, equity (the likelihood to reduce inequities or increase equity) and resource implications (resource intensity) of the intervention ((12,13)). In alignment with the GRADE approach, this systematic review aimed to assess the domains of acceptability, feasibility, equity and resource use for prenatal screening for CT and NG. **Table 1** shows the eligibility criteria for study selection.

**Table 1:** Eligibility criteria

Criteria	Description
Population	Pregnant adults and adolescents (12 years of age and older, up to and including delivery)
Intervention(s)	A screening strategy involving: · Nucleic Acid Amplification Test (NAAT) for CT and NAAT or culture for NG · Urine, vaginal, or cervical samples for NAATs; urethral or endocervical samples for cultures · A universal or targeted approach · Any timing (i.e., the point during pregnancy at which the screening test is performed) · Any frequency (i.e., number of times the screening test is conducted during pregnancy) · Any subsequent management of pregnant persons with confirmed infection, including no active management
Comparator(s)	An alternative screening strategy conducted with an alternative test, specimen, approach, timing, different frequencies, any subsequent management strategy for pregnant persons with confirmed infection (including no management), as well as no screening strategy
Outcome(s)	Studies should assess one or more of the following factors: · Acceptability of any strategy to screen for CT or NG during pregnancy from the perspective of any stakeholder · Feasibility/quality of implementation of any strategy to screen for CT or NG during pregnancy · Cost/resources or cost effectiveness · Equity of any strategy to screen for CT or NG during pregnancy including socioeconomic status, age, race/ethnicity, religion, geographical location (urban/rural), education level, income level and health insurance coverage o The following definition of equity by the World Health Organization (WHO) ((15)) was used for this systematic review: “the absence of avoidable, unfair, or remediable differences between groups of people, whether those groups are defined socially, economically, demographically or geographically or by other means of stratification. Health equity or equity in health implies that ideally, everyone should have a fair opportunity to attain their full health potential and that no one should be disadvantaged from achieving this potential”
Types of studies	Any study design, except for the following: case studies, case reports of an individual patient, letters, commentaries, opinion pieces and editorials
Type of setting	Studies conducted in Australia, Canada, the European Economic Area, New Zealand, the United Kingdom or the United States of America
Timeframe	Studies published between January 1, 2003, and January 14, 2021

Abbreviations: CT, *Chlamydia trachomatis*; NG, *Neisseria gonorrhoeae*

### Information sources

Studies were identified by searching electronic databases, scanning reference lists of included articles and consulting subject matter experts from the NAC-STBBI. The CDA-AMC HTA report on screening for CT and NG during pregnancy, consisting of a review of the clinical literature, an economic analysis and a review of qualitative studies on patients’ preferences and experiences ((11)), was also reviewed to identify relevant studies. In consultation with an external methodology expert, the GRADE search strategy tool (not yet validated) for identifying published literature on acceptability, feasibility, equity and resource use was modified to avoid limiting the search by country. During screening, studies conducted in countries comparable to Canada’s healthcare context were included in the review.

A Health Canada librarian incorporated the modified GRADE search strategies within the original CDA-AMC HTA clinical review search strategy. The MEDLINE search strategy was reviewed by the evidence review team. MEDLINE® All, Embase and the Cochrane Central Register of Controlled Trials (Cochrane CENTRAL) were searched on the Ovid platform from 2003 to present (January 14, 2021). The search start year of 2003 was informed by PHAC’s laboratory diagnosis recommendations of STIs ((16)). No study design limit was applied and language was limited to English or French. The search strategies for the three databases are presented in **Appendix, Supplemental material, Appendices A to F**. Results from the original search were exported on September 19, 2019, and results from the update search were exported on January 14, 2021 (to identify any relevant new studies published since June 1, 2019). RefWorks was used to remove duplicates and store the citations. Microsoft Excel databases were used to record the process.

### Study selection and data extraction

For the original search, the number of retrieved records was split among three individuals and screened by title and abstract based on inclusion criteria. For the update search, the retrieved records were independently screened by two individuals. For both searches, any differences were resolved through discussion between the reviewers or in consultation with another individual. Any uncertainty in the inclusion of titles and abstracts led to the retrieval of the full text article.

Any full text articles that were not available online were retrieved via the PHAC library. For the original search, the number of selected full text articles was split among three individuals and assessed based on inclusion criteria, which were then verified by another individual. For the update search, one individual assessed the selected full text articles based on inclusion criteria, which were then verified by two individuals. For both searches, any differences were resolved through discussion between the reviewers and in consultation with another individual.

A data extraction form was developed, pilot-tested on two randomly selected included studies and revised accordingly. Reviewers were trained on extracting data using the form by the primary author. For the original search, the number of articles that met the inclusion criteria was split among three individuals who then extracted data and another individual verified the extracted data. For the update search, an individual extracted data from the articles that met the inclusion criteria and two individuals verified the extracted data. The information extracted from each study included study design, study funding source, number of participants, participant age, race/ethnicity, study duration, country where the study was conducted, setting, intervention(s) and results on acceptability, feasibility, equity and resource use. The data extraction form template is presented in Supplemental material, **Appendix G**.

### Quality assessment

The Risk of Bias Assessment Tool for Non-Randomized Studies (RoBANS) was used for quality assessment of the included observational studies ((17)). The RoBANS tool consists of six domains and a judgment of “high”, “low” or “unclear” can be assigned to each domain. Each included study was assessed for risk of bias by a reviewer and another reviewer verified the assessments.

### Synthesis of evidence

A narrative synthesis of the included studies was performed for this review. Findings were presented by acceptability, feasibility, equity, resource use or combination thereof.

## Results

Supplemental material, **Figure S1** shows the flow diagram of study selection. Of the 1,386 records (original search=1,226 and update search=160) identified through searching electronic databases and reviewing the CDA-AMC HTA report ((11)), 12 articles (original search=9 and update search=3) met the inclusion criteria and were included in this systematic review. The combined results from the original search and the update search are presented herein.

Supplemental material, **Table S1** displays the characteristics and findings of the included studies on acceptability, feasibility, equity and resource use. The study designs were cross-sectional, retrospective chart reviews and economic evaluations. The studies were conducted in Australia, Canada, the Netherlands, the United Kingdom and the United States. Supplemental material, **Table S2** shows the risk of bias assessment findings for each included observational study. The quality of the included articles was generally strong. Selection bias was “high” for eight studies. Four studies did not report on sources of funding and three studies did not report on competing interests.

### Acceptability

Four studies reported on acceptability of prenatal screening for CT or NG. Logan *et al.* compared screening approaches to identify CT in a sample of 209 miscarriage individuals at a hospital in Scotland, United Kingdom ((18)). Among participants, a urine sample was significantly preferred over vulval swab (*p*<0.0001) or endocervical swab (*p*<0.0001). A vulval swab was significantly preferred compared to an endocervical swab (*p*<0.0001). However, there was reduced test performance with urine sample. The reasons for declining the endocervical method were categorized into the following themes: physically negative aspects, positive aspects of non-invasive testing, not wishing to repeat an internal exam, feeling psychologically unable to cope with the procedure and the impact of the screening procedure on the pregnancy.

As part of a larger study assessing the prevalence and factors associated with CT in pregnancy ((19)), Bilardi *et al.* examined the acceptability of screening for CT in 100 pregnant persons aged 16–25 years at four major antenatal services across Melbourne, Australia ((20)). The researchers found that all participants supported testing for CT as part of their routine antenatal care and nearly all strongly preferred urine testing compared to the other methods, as it was quick, easy and non-invasive. The main motivating factor in the acceptability of screening was concern for the health of the baby and the main concern expressed was whether testing and treatment could potentially harm the baby.

Pereboom *et al.* assessed knowledge, attitudes and experiences of CT screening in 383 pregnant persons and 282 partners at 22 primary midwifery care practices in the Netherlands ((21)). In this study, 347 (54.2%) pregnant persons and partners reported that all pregnant people should routinely be tested for CT in antenatal care and 85 (13.3%) reported that only those at increased risk should be tested. The researchers found that 3.7% of pregnant persons and 1.8% of partners felt stigmatized and 2.7% of pregnant people and 1.1% of partners felt ashamed by having a CT test offered.

Vainder *et al.* assessed prenatal screening for NG and CT in 1,220 pregnant persons at an urban tertiary care centre in Ontario, Canada ((8)). Of the 733 individuals with a record of testing method, 92.0% were tested by urine and 8.0% by cervical swab. There was no statistically significant difference in the testing rates among midwives (93.8%), family physicians (91.4%) and obstetricians (88.5%).

### Feasibility and equity

Four articles reported on feasibility and equity for prenatal screening for CT or NG. Miller *et al.* (2003) assessed NG in 751 pregnant persons attending a community-based prenatal program in an underserved area in Louisiana, United States ((22)). The researchers found that among pregnant individuals aged ≤19 years, 23 (7.2%) were positive in the initial testing and 11 (3.5%) were positive only in the later testing. Among those aged ≥20 years, 15 (3.5%) were positive in the initial testing and 8 (1.8%) were positive only in the later testing.

Miller *et al.* (2005) examined identifying CT through initial versus repeat screening in 752 pregnant persons attending a community-based prenatal program in an underserved area in Louisiana, United States ((23)). The researchers found that at the time of initial testing, pregnant individuals aged ≤19 years had significantly higher rates of CT compared to those aged ≥20 years (odds ratio [OR] 2.19; 95% CI: 1.44–3.23; *p*<0.001). Among those with an initial negative test, pregnant individuals aged ≤19 years had significantly higher rates of CT compared to those aged ≥20 years at 34-week follow-up testing (OR 4.24; 95% CI: 1.85–9.74; *p*<0.001). Eight infections would have been missed if repeat testing had been limited to those aged ≤19 years.

Chen *et al.* assessed risk factors associated with CT and the sensitivity and specificity of these when used for selective screening in 987 pregnant persons aged 16–25 years at four major antenatal services across Melbourne, Australia ((19)). The researchers found that having more than one sexual partner in the past year was associated with CT infection (adjusted OR 11.5; 95% CI: 7.1–18.5). They noted that screening restricted to pregnant persons who reported more than one sexual partner in the past year would have detected 44% of CT in those aged 16–25 years and would have required only 7% of individuals to be screened. The addition of pregnant persons aged ≤20 years would have required 27% to be screened and detection of 72% of CT.

Leichliter *et al.* assessed receipt of CT screening in the past 12 months in 1,155 people who were pregnant in the past 12 months or at time of interview in the United States ((24)). The researchers found that those who reported receiving prenatal care were significantly more likely to receive CT testing than individuals who had not received prenatal care (adjusted OR 2.10; 95% CI: 1.35–3.28). People living in other areas of a metropolitan statistical area were significantly less likely to receive CT testing than those living in the principal city of an metropolitan statistical area (adjusted OR 0.62; 95% CI: 0.44–0.86). People who were born outside of the United States were also significantly less likely to receive CT testing than those who were born in the United States (adjusted OR 0.35; 95% CI: 0.19–0.64).

### Feasibility and resource use

One observational study and three economic evaluations reported on feasibility and resource use of prenatal screening for CT or NG. Tyker *et al.* examined screening for CT and NG in 102 pregnant persons aged 13–19 years at an adolescent obstetrics practice in Ontario, Canada ((25)). Urine Nucleic Acid Amplification Test (NAAT) was used for 88 of 89 (98.9%) patients screened in the third trimester. The researchers noted that the decision to use urine samples was based on feasibility and ease of collecting samples, whereas using an endocervical swab in the third trimester is more resource intensive and invasive.

Ong *et al.* assessed the cost effectiveness of screening all pregnant persons aged 16–25 years for CT compared with selective screening or no screening using a 12-month time horizon and from a third-party payer perspective, in Australia ((26)). With a CT prevalence estimate of 3%, screening all pregnant persons aged 16–25 years during their first antenatal visit compared to no screening was cost-effective, as it would cost the health system 1,641 Australian dollars (AUD) per CT case detected and treated, and 34,931 AUD per quality-adjusted life year (QALY) gained. Screening all pregnant persons aged 16–25 years compared to no screening would have cost savings when CT prevalence was above 11%. With a CT prevalence estimate of 3%, screening all pregnant persons aged 16–25 years compared to selective screening would cost the health system 5,448 AUD per CT case detected and treated, and 116,213 AUD per QALY gained. Screening all pregnant persons aged 16–25 years was cost-effective compared to selective screening when CT prevalence was above 5%.

Rours *et al.* analyzed the cost effectiveness of antenatal screening of all pregnant persons for CT from a societal perspective (inclusion of non-medical [indirect] costs due to production losses) in the Netherlands ((27)). In the base-case analysis, they estimated 527,900 euros (EUR) to detect and treat CT for 1,000 pregnant persons and their partners, and averted medical costs were estimated at 626,800 EUR. In sensitivity analysis, the net cost savings remained with test costs up to 22 EUR (test price: 19 EUR) for a range of underlying assumptions. In scenario and probabilistic analyses, the cost savings increased with targeted screening of pregnant persons aged ≤30 years or with first pregnancies only.

Ditkowsky *et al.* (2017) assessed the cost-benefit of screening all pregnant persons aged 15–24 years for CT compared with no screening using a 12-month time horizon and from a third-party payer perspective in a high burden setting in the United States ((28)). Screening was proven to offer net cost savings when prevalence estimates were above 16.9%. At the prevalence estimate of 6.7%, there was an estimated net increase in expenditure of 142,66 million US dollars (USD) (22.14 USD/individual) with 204,630 cases of treated CT.

## Discussion

This is the first systematic review on acceptability, feasibility, equity and resource use for prenatal screening for CT and NG. Nine observational studies reporting on approximately 5,000 participants and three economic evaluations were included in this review.

In general, pregnant persons and healthcare providers accepted prenatal screening for CT and NG. Most pregnant persons and partners supported testing of all pregnant individuals for CT as part of routine antenatal care. Some pregnant persons and partners reported feelings of stigma and shame when offered testing for CT. Similarly, Pavlin *et al.* found that barriers to acceptance of CT testing among women in general include denial of risk of infection; stigma associated with a positive diagnosis; feelings of shame, guilt, embarrassment, anger, fear and anxiety; concerns around privacy and confidentiality; time; and sample collection method ((29)).

Pregnant persons preferred non-invasive sampling approaches compared to other methods. Similarly, Oakeshott *et al.* found that among pregnant persons with less than 10 weeks of gestation, 47% preferred urine, 5% preferred self-collected vulval swab and 48% indicated no preference ((30)). In addition, Pimenta *et al.* found that pregnant persons aged 16–24 years preferred urine screening over cervical or vaginal swabs taken by healthcare providers across a variety of healthcare settings ((31)).

In terms of feasibility and equity, persons who did not receive prenatal care and individuals born outside of the United States were less likely to receive CT testing compared to their counterparts. These findings may have been underestimated if CT testing during pregnancy had occurred outside the survey timeframe of the past 12 months. These findings are also generally in alignment with literature showing inequities in access to prenatal care in Canada. Findings from the Maternity Experiences Survey ((32)) in mothers aged ≥15 years showed that the prevalence of inadequate prenatal care was 18.9% in Canada, with the highest estimates in Nunavut (28.8%) and the Northern Territories (24.9%). In addition, mothers who were immigrants were more likely to receive inadequate prenatal care compared to individuals born in Canada (OR 1.40; 95% CI: 1.13–1.74).

Individuals who were pregnant in the past 12 months and living outside of the principal city of metropolitan statistical areas (e.g., suburban area) were less likely to receive CT testing compared to those living in other areas. This finding is in slight contrast to evidence showing that pregnant individuals living in rural or remote areas may not always have access to trained prenatal healthcare providers in Canada ((33)). Evidence on pregnant persons with high risk for and a high prevalence of CT and NG from an underserved area in the United States showed that, if repeat screening was limited to individuals aged ≤19 years, eight cases could have been missed among those aged ≥20 years. This finding highlights how targeted screening could miss cases in those who do not meet the screening criteria and that limiting screening to earlier in pregnancy could potentially miss detecting new infections and reinfections ((11)).

With regard to resource use, screening all pregnant persons compared to no screening has cost savings. In general, the studies showed that an increase in the prevalence of CT and NG infections contributes to better cost-effectiveness.

## Limitations

The included studies have several limitations to consider when interpreting the findings. Firstly, some of the observational studies were conducted in a miscarriage sample, younger age groups or those with a high risk for and a high prevalence of CT and NG that could contribute to selection bias. The findings from these studies may not be generalizable to the larger population of pregnant persons and those with lower prevalence of CT or NG. Secondly, some of the observational studies used self-report questionnaires (e.g., self-reported CT testing) that could potentially introduce recall bias. Thirdly, the economic evaluations focused on CT only. In addition, two of these studies were limited to younger age groups, a 12-month time horizon and a third-party payer perspective ((26,28)). One study was conducted in a higher burden setting and the researchers noted possible uncertainty in the estimated rates of CT-related sequelae that could contribute to overestimating the cost savings of CT screening ((28)). The strengths of the studies included in this review were the use of semi-structured interviews and the inclusion of a variety of healthcare settings.

This systematic review did not identify eligible literature on acceptability, feasibility, equity and resource use of timing of repeating universal screening (e.g., third trimester or at delivery). It also did not identify eligible literature on acceptability of prenatal screening for NG among partners of pregnant persons. These gaps in the literature highlight areas for future research.

The strengths of this review include the incorporation of the GRADE search strategies on acceptability, feasibility, equity and resource use and inclusion of different types of studies.

## Implications

The evidence from this systematic review supported the development of the updated NAC-STBBI recommendations on prenatal screening for NG and CT in Canada ((34)). Screening all PWPI at first and third trimesters is likely more acceptable than targeting high-risk PWPI because it may reduce the stigma associated with screening for an STI. A recommendation about the sampling method for screening was not made since the preference and capacity may vary according to the individual, healthcare provider and the healthcare system. The updated NAC-STBBI recommendations are as follows ((34)):

· We suggest screening all PWPI for NG and CT during the first trimester or at the first antenatal visit and again in the third trimester (conditional recommendation; low certainty evidence)

· We suggest screening PWPI at the time of labour for NG and CT in any of the following situations (conditional recommendation; low certainty evidence):

· No prenatal screening has occurred (no valid results available at the time of labour)

· Third trimester screening has not occurred

· A positive test result was obtained for NG or CT during pregnancy without appropriate follow-up, including treatment and a test-of-cure

## Conclusion

In general, prenatal screening for CT and NG is acceptable among pregnant persons and healthcare providers. Most pregnant persons and partners supported testing of all pregnant individuals for CT as part of routine antenatal care. Inequities in feasibility (accessibility to screening) exist in certain populations. Studies have shown that targeted screening can miss cases. Screening all pregnant persons for CT has net cost savings compared to no screening in the included studies. More comparative research is needed on acceptability, feasibility, equity and resource use for prenatal screening for CT and NG in the Canadian context. These findings were used to support the updated NAC-STBBI recommendations on prenatal screening for CT and NG.

## Appendix

Supplemental tables, figure, search strategies and template for data extraction form are available upon request to the corresponding author: shamila.shanmugasegaram@phac-aspc.gc.ca

Appendix A: Database(s): Ovid MEDLINE® ALL 1946 to September 17, 2019

Appendix B: Database(s): Embase 1974 to September 18, 2019

Appendix C: Database(s): EBM Reviews - Cochrane Central Register of Controlled Trials August 2019

Appendix D: Database(s): Ovid MEDLINE® ALL 1946 to January 13, 2021

Appendix E: Database(s): Embase 1974 to January 13, 2021

Appendix F: Database(s): EBM Reviews - Cochrane Central Register of Controlled Trials November 2020

Appendix G: Template for Data Extraction Form - Acceptability, Feasibility, Resource Use and Equity of NG/CT Screening During Pregnancy

Table S1: Study characteristics and findings on acceptability, feasibility, equity and resource use for prenatal screening for CT and NG

Table S2: Quality assessment of included studies

Figure S1: Flow diagram of study selection for original and update searches
